# Ultra-High-Speed Accelerator Architecture for Convolutional Neural Network Based on Processing-in-Memory Using Resistive Random Access Memory

**DOI:** 10.3390/s23052401

**Published:** 2023-02-21

**Authors:** Hongzhe Wang, Junjie Wang, Hao Hu, Guo Li, Shaogang Hu, Qi Yu, Zhen Liu, Tupei Chen, Shijie Zhou, Yang Liu

**Affiliations:** 1 State Key Laboratory of Electronic Thin Films and Integrated Devices, University of Electronic Science and Technology of China, Chengdu 610054, China; 2 School of Materials and Energy, Guangdong University of Technology, Guangzhou 510006, China; 3 School of Electrical and Electronic Engineering, Nanyang Technological University, Singapore 639798, Singapore; 4 School of Information and Software Engineering, University of Electronic Science and Technology of China, Chengdu 610054, China

**Keywords:** RRAM, convolutional neural network, high-speed target detection, quantization

## Abstract

Processing-in-Memory (PIM) based on Resistive Random Access Memory (RRAM) is an emerging acceleration architecture for artificial neural networks. This paper proposes an RRAM PIM accelerator architecture that does not use Analog-to-Digital Converters (ADCs) and Digital-to-Analog Converters (DACs). Additionally, no additional memory usage is required to avoid the need for a large amount of data transportation in convolution computation. Partial quantization is introduced to reduce the accuracy loss. The proposed architecture can substantially reduce the overall power consumption and accelerate computation. The simulation results show that the image recognition rate for the Convolutional Neural Network (CNN) algorithm can reach 284 frames per second at 50 MHz using this architecture. The accuracy of the partial quantization remains almost unchanged compared to the algorithm without quantization.

## 1. Introduction

Research on Processing-in-Memory (PIM) has been extensively carried out [1,2,3,4,5,6]. Various memory devices, including SRAM, DRAM, floating gate memory, etc., have been utilized in PIM architectures. Recently, Resistive Random Access Memory (RRAM) has been proposed for PIM architectures due to its compact cell structure, simple processing technology, and low power consumption [7,8,9,10,11,12,13,14,15]. RRAM crossbar arrays are commonly used for in-memory computation [16,17,18]. DACs and ADCs are typically used for the data conversion of input and output [19,20,21]. However, when applying complex Convolutional Neural Network (CNN) algorithms such as VGGNet, GoogLeNet, ResNet, and You-Only-Look-Once (YOLO) series, high-speed and high-precision Analog Digital Converters (ADCs) and Digital Analog Converters (DACs) are necessary for data conversion. Consequently, a substantial amount of power consumption and a large chip area are expected [22].

In this work, we propose an RRAM PIM architecture that eliminates the need for ADCs and DACs for data conversion. There is no additional memory usage in convolution computation. The main contributions of this article include:

1. Introducing an RRAM PIM architecture that can achieve the high-speed processing of CNN without requiring DACs and ADCs;
2. Proposing a partial quantization scheme that can reduce accuracy loss;
3. Achieving an acceleration of up to 284 frames per second while maintaining almost the same accuracy as that without quantization.

**Figure 1:**
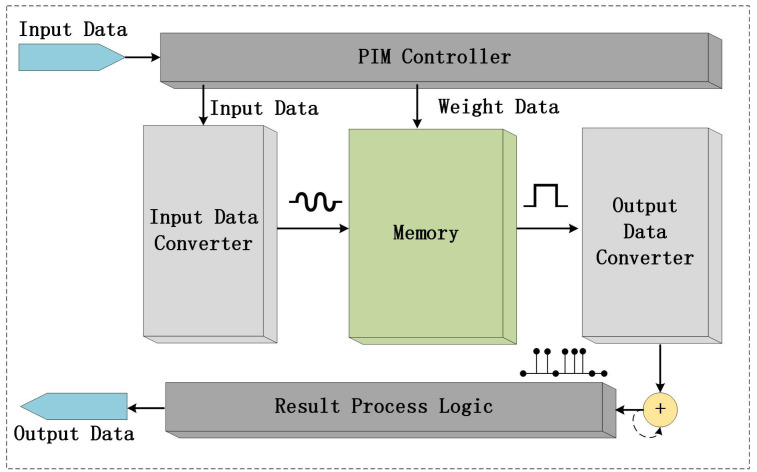
Schematic illustration of a conventional PIM configuration.

A conventional PIM configuration is illustrated in Figure 1. The design includes a PIM controller, input data converter, output data converter, and memory and result processing module. The memory array can be implemented using various memory devices such as Flash, SRAM, RRAM, among others. The PIM controller first writes weight data into memory array, and the data are then converted by the converter. The conversions may involve transforming the data from column to row and from digital to analog format. The converted data are then processed inside memories with weight data. If the in-memory processes are carried out in the analog domain, the output data should be converted back into the digital domain using an ADC. The digital signals are then transmitted to the next round of computation.

**Figure 2:**
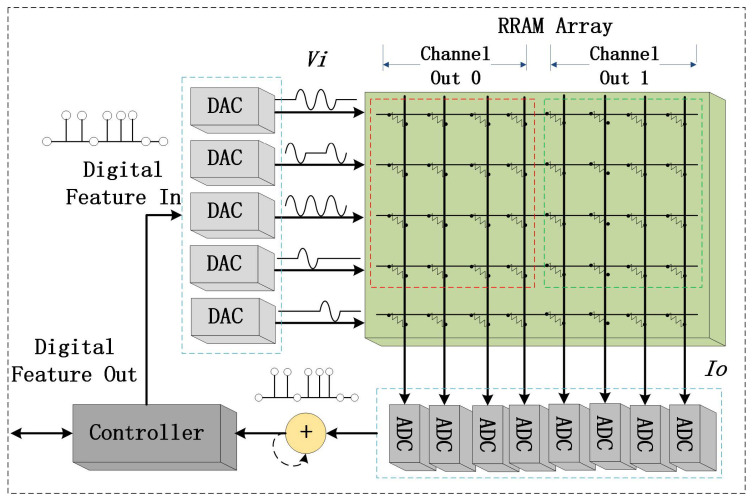
Schematic illustration of a typical RRAM PIM configuration.

A typical RRAM PIM configuration is depicted in Figure 2. The design mainly includes a DAC, RRAM array, ADC, and system controller [23]. After the systemic initialization by the controller, the DAC array converts features from digital to analog to enable processing in RRAM. The RRAM array then performs computations on these data within the memories in the analog domain. The resulting analog signals are output from the RRAM and should be converted back into digital by the AD, after which they are processed by the controller in the next step, as shown in Figure 2.

## 2. Methods

### 2.1. Architecture

**Figure 3:**
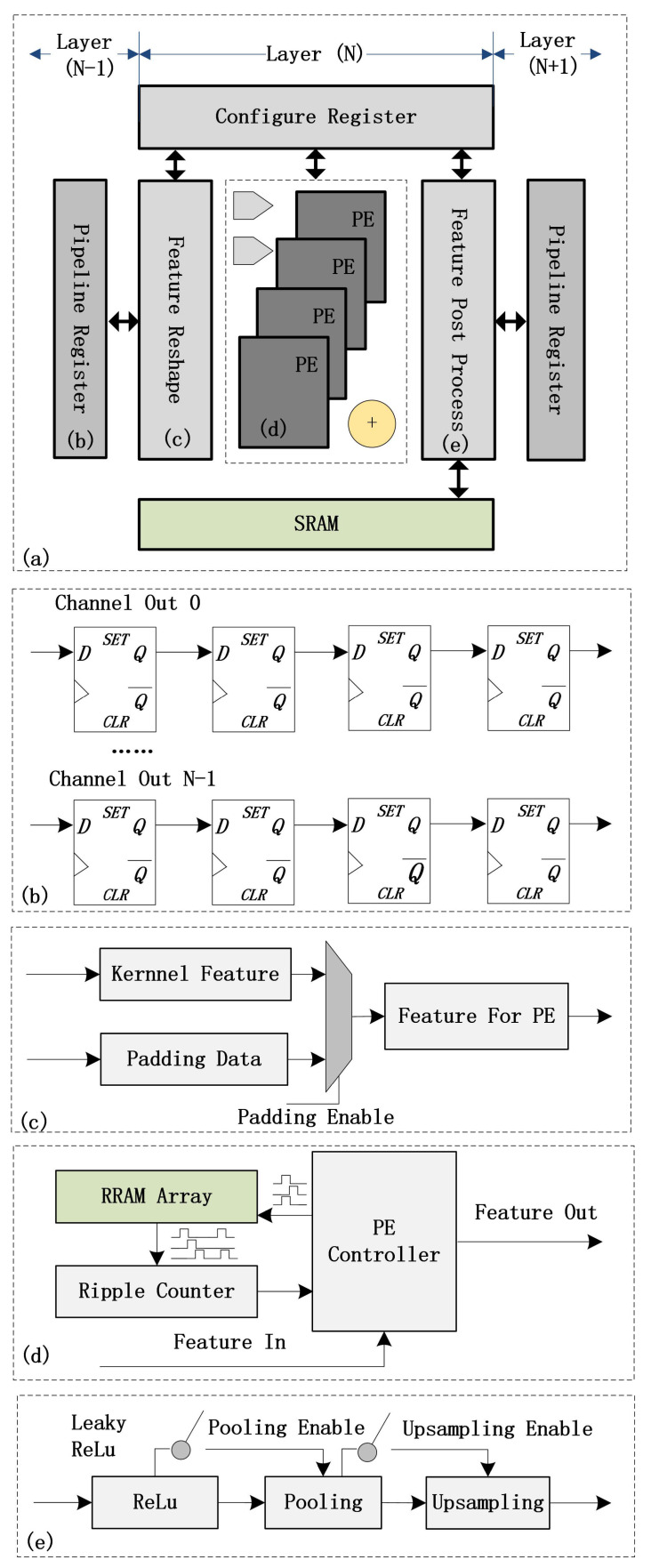
(**a**) Schematic illustration of the proposed RRAM PIM architecture; (**b**) The architecture consists of the pipeline register; (**c**) feature-reshaping module; (**d**) PE array; and (**e**) Post-feature processing module.

The conventional RRAM computing accelerator comprises RRAM arrays, control modules, ADCs, and DACs [23], which is a suitable technique for the lightly-scaled algorithm. However, as the scale of the algorithm increases, the power consumption and chip area required for the ADCs and DACs increase significantly. The proposed RRAM PIM architecture, shown in Figure 3a, consists of the pipeline register (Figure 3b), feature-reshaping module (Figure 3c), Process Element (PE) array (Figure 3d), post-feature processing module (Figure 3e), configuration register, and SRAM module. The output feature from Layer (N-1) is stored in the pipeline register. Then, the feature shape is modified in the feature-reshaping module. The feature after reshaping is sent to the RRAM array, and convolution computation is performed with the weight matrix stored in the RRAM array. After the post-feature processing, including biasing, activation, pooling operation, etc., the result is sent to the pipeline register for computation in the next layer.

- The Pipeline Register is shown in Figure 3b. The input is from Layer (N-1). As the configuration no longer needs to be written to memory, the timing feature is also required for the next layer;
- Feature Reshaping module is shown in Figure 3c. For rows or columns that require padding, “0” or “1” in the feature is stored according to the padding request. After feature reshaping, the feature in a kernel is flattened and sent to the RRAM operation core in the PE module.
- The Process Element is shown in Figure 3d. The PE is implemented based on the RRAM array. To reduce hardware consumption for large-scale algorithms, this design does not utilize ADCs and DACs. D-flip-flop and ripple counter are used to convert features into pulses. The features are input to the RRAM array according to the bit order. All the computations are conducted in the digital domain to reduce the conversion cost;
- The post-feature processing module is shown in Figure 3e. Activation and biasing are processed inside the module. The “ReLU” activation function is used, but it is possible to implement other activation functions suitable for algorithms other than CNN.

### 2.2. Partial Quantization

**Figure 4:**
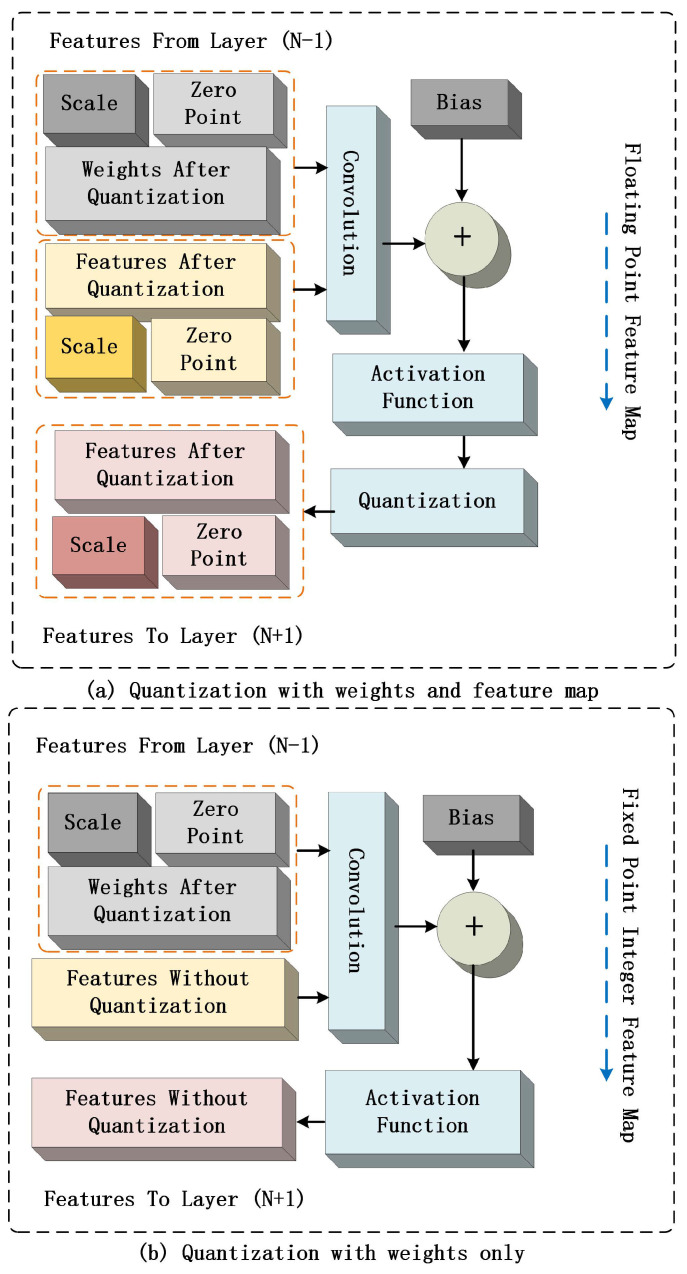
Schematic illustration of (**a**) full quantization (i.e., quantization of both weights and features) and (**b**) partial quantization (i.e., quantization of weights only) of convolutional neural network.

In this work, a partial quantization technique is proposed in conjunction with the characteristics of RRAM PE. The quantization of weights can substantially reduce the RRAM storage space. The weights for use were trained in the Pytorch^®^ platform. As shown in Figure 4a, both the weights and the features are quantized, but such full quantization may lead to accuracy loss. In contrast, Figure 4b shows a partial quantization where only the weights are quantized. After training, the floating-type data are quantized into three parts: 8-bit fixed-point integer for weights, 32-bit floating-point digit for scale factor, and 8-bit fixed-point integer for zero-point parameters. During computation, the 8-bit fixed-point integer weights are written to the RRAM weight matrix, and the scale factor and zero-point parameters are mapped to 25-bit fixed-point digits.

The Weight matrix is quantized and dequantized according to Equation (1),

(1) $$y_{\left(cout,x,y\right)}=\sum_{c=0}^{CIN-1}\sum_{i=0}^{KX-1}\sum_{j=0}^{KY-1}x_{\left(c,x+i,y+j\right)}\cdot \left(w_{\left(cout,c,i,j\right)}-zp_{w}\right)\cdot scale_{w}+bias_{cout}$$

where $x_{\left(c,x+i,y+j\right)}$ is the feature with its input channel; $w_{\left(cout,c,i,j\right)}$ is a weight with the channel; $zp_{w}$ represents the zero points of weight in the channel; $scale_{w}$ is the scaling factor; and $bias_{cout}$ is the bias with the channel.

If the zero points of weights can be trained to zero, Equation (1) can be further simplified as follows:

(2) $$y_{\left(cout,x,y\right)}=\sum_{c=0}^{CIN-1}\sum_{i=0}^{KX-1}\sum_{j=0}^{KY-1}x_{\left(c,x+i,y+j\right)}\cdot w_{\left(cout,c,i,j\right)}\cdot scale_{w}+bias_{cout}$$

For the feature-out module, since the operation of writing memory is not required, it is possible to transfer the feature out to the next layer in the 16-bit fixed-point integer without quantization.

### 2.3. Data Flowing without Additional Memory Storage

**Figure 5:**
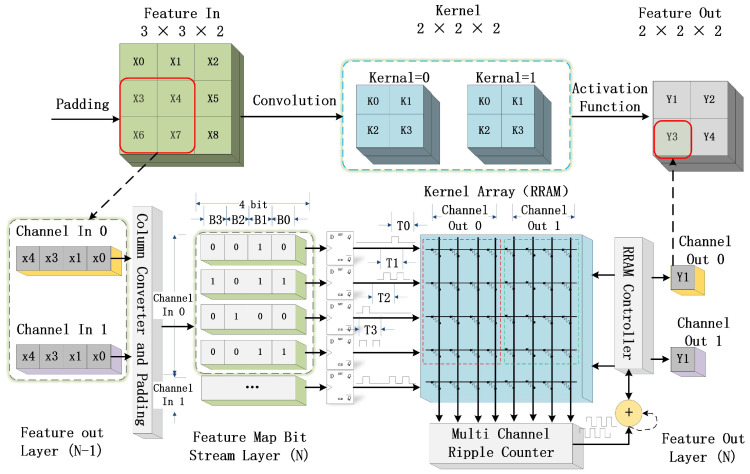
Illustration of data flow in the proposed RRAM PIM architecture.

To reduce the data conversion cost, the ADCs and DACs are not utilized in this design. Instead, the ripple counter in conjunction with the RRAM array can effectively conduct convolution without data conversion, as demonstrated in Figure 5.

As shown in Figure 5, the RRAM PE unit comprises a column converter unit, a padding processing unit, a multi-bit D-flip-flop register, a RRAM computing matrix, a ripple counter, and a controller. As an example, for the input feature with a 3 × 3 × 2 matrix, the output is a 2 × 2 × 2 feature for the convolution with two 2 × 2 × 2 kernels.

The column converter and the padding unit reshape the input feature. This feature vector is sequentially sent into the RRAM matrix in column order by the D flip-flop register. The column vector is input to the RRAM matrix in row order. That is, we first obtain a column vector, in time $T_{0}$; we send $X_{in}$[0][0] to Row 0 of the RRAM array; and the ripple counter counts the output pulse of each column. At time $T_{1}$, $X_{in}$[1][0] will be sent to the RRAM array; and the ripple counter continues to count the pulse output of each column and accumulate the pulse amount. After all the row bits have been sent, a step of computation is completed.

(3) $$T_{win}=B_{x}\cdot C_{in}\cdot \left(K_{x}\cdot K_{y}\right)$$

The time to complete a window feature $T_{win}$ can be determined using Equation (3), where $C_{in}$ is the input feature; $B_{x}$ is the bit width of $X_{in}$; $K_{x}$ is the kernel size in the x-direction; and $K_{y}$ is the kernel size in the y-direction. $K_{x}$ and $K_{y}$ are determined by the algorithm. They can be considered as coefficients. Hence $B_{x}$ and $C_{in}$ determine the computation time of the window. By selecting the appropriate bit width, and if the number of input channels is supported by the RRAM array, the operation speed of the entire system can be further increased.

### 2.4. Mapping of Algorithm to the Proposed RRAM PIM

To demonstrate the feasibility of the proposed RRAM PIM, the YOLOv3 algorithm is used for mapping. The YOLOv3 algorithm includes a ResNet Block and YOLO Block. The main operator contains convolution, leaky ReLU, up-sampling, route, and common pooling operator. The operations are consolidated by a convolution with a kernel size of 3 × 3 and stride of 2 × 2.

To increase the processing speed, a large convolution computation can be distributed to a plurality of PE units. The computation of the PE units is carried out in parallel. The computation results of all the PE units are then accumulated together. When all the PE units have completed their computations, the convolution result is obtained. This result is then sent to the activation function of the post-feature processing module, up-sampling module, etc. The output feature is stored in the Pipeline Register and used as the input feature for the next-layer computation.

To improve the performance of the accelerator, multiple PEs are used for parallel computations. Each PE is responsible for computing a column of features, effectively reducing the computation time for each layer.

In the ResNet block of YOLOv3 algorithm, the output features from all the previous layers are accumulated to produce the input feature of the current layer. This requires the storage of the output feature of each of the previous layers, which is stored in SRAM as shown in Figure 3. No other memory is required in the architecture.

### 2.5. Au/Ni/HfO_2_/Ni RRAM Model

**Figure 6:**
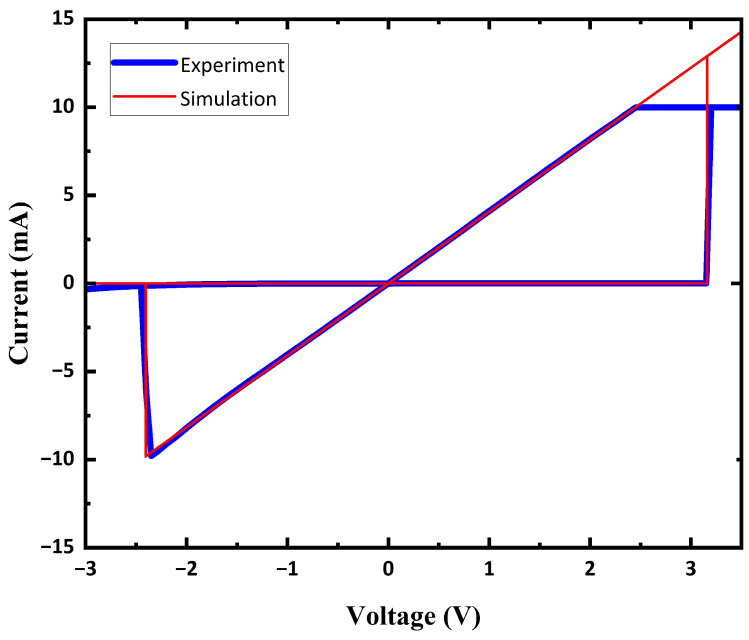
Electrical characteristics of Au/Ni/${\mathrm{HfO}}_{2}$/Ni RRAM obtained from experiment and simulation.

Figure 6 shows the electrical characteristics of the Au/Ni/${\mathrm{HfO}}_{2}$/Ni RRAM (blue line), which is reported in our previous work [24]. The resistance of the RRAM can be substantially changed by applying a voltage that exceeds its threshold. When a positive voltage is applied through RRAM (from top-electrode to bottom-electrode) and exceeds its positive threshold ($V_{\mathrm{SET}}$), the resistance of RRAM decreases to the low-resistance state (LRS); this process is generally called the SET process. When a negative voltage is applied through RRAM (from bottom-electrode to top-electrode) and exceeds its negative threshold ($V_{\mathrm{RESET}}$), the resistance of RRAM increases to the high-resistance state (LRS); this process is generally called the RESET process.

The RRAM model that is adapted from the Knowm’s mean metastable switch (MMSS) memristor model [25] is used to fit the experimental data of the Au/Ni/${\mathrm{HfO}}_{2}$/Ni RRAM. The equation for the Au/Ni/${\mathrm{HfO}}_{2}$/Ni RRAM model is as follows:

(4) $$G\left(X\right)=\frac{S(x)}{R_{ON}}+\frac{S(1-x)}{R_{OFF}}$$

(5) $$S\left(X\right)=\frac{1}{1+{\left(\frac{X}{1-X}\right)}^{\alpha }}$$

(6) $$\frac{dX}{dt}=\frac{1}{\tau }\left[\frac{1}{1+e^{-\beta (V-V_{ON})}}\cdot \left(1-X\right)-\left(1-\frac{1}{1+e^{-\beta (V+V_{OFF})}}\right)\cdot X\right]$$

where G(x) represents the conductance of the RRAM. *X* represents the state variable of the RRAM, which is a value between 0 and 1. When X=0, G(X) reaches its minimum value. When X=1, G(X) reaches its maximum value. $\frac{dX}{dt}$ represents the drift velocity of the state variable, where τ is a time constant, and $\beta =\frac{q}{kT}={\left(V_{T}\right)}^{-1}$. $V_{T}$ represents the thermal voltage, where *q* is the elementary charge, *k* is the Boltzmann constant, and *T* is the temperature. $V_{ON}$ and $V_{OFF}$ represents the positive and negative thresholds of the RRAM, respectively. S(x) is an S-shape curve function, where α is the tuning constant to adjust the nonlinearity of the S(X) function (S(0.5)=0.5, $\lim_{X\to 0}S\left(X\right)=0$, $\lim_{X\to 1}S\left(X\right)=1$, α<0). By introducing the S(x) function, the hard-switching characteristics of the Au/Ni/${\mathrm{HfO}}_{2}$/Ni RRAM can be better fitted. The simulated characteristics of the Au/Ni/${\mathrm{HfO}}_{2}$/Ni RRAM is shown in Figure 6 (red line), and the parameters that are used in this work are listed in Table 1.

## 3. Results and Discussion

**Figure 7:**
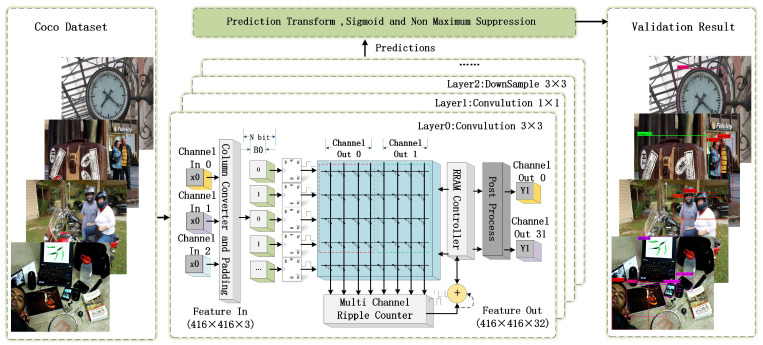
Illustration of image identification using the proposed RRAM PIM.

The internal delay can be obtained through the SPICE^®^ simulation. The hardware acceleration platform of YOLOv3 is used. The system was designed using Verilog except for the RRAM model. The working frequency was evaluated using the Design Compiler (DC) and IC Compiler (ICC) tools. Au/Ni/${\mathrm{HfO}}_{2}$/Ni RRAM arrays were modeled by Verilog-A, which is a non-synthesizable Verilog construct. In the proposed design, only two states are required for RRAM (i.e., HRS and LRS), and the ON/OFF ratio of Au/Ni/HfO${}_{2}$/Ni RRAM is large enough. Thus, it is applicable to utilize an idealized RRAM model to simulate the proposed PIM architecture. The size of each RRAM array in the proposed design is 36 × 256, which contains 9216 RRAM devices. The data are fed into the RRAM array in series, while the outputs are read in parallel (32 × 8 bits). VCS2018^®^ of Synopsis is used for the compiling and for the simulation of the architecture. The testbench is built using SystemVerilog and Universal Verification Methodology (UVM^®^); 5000 images from COCO datasets [26] are selected for identification. The images from the COCO dataset are converted to binary files that were read by SystemVerilog and sent to the proposed system (DUT). The testbench monitored the intermediate results and the data saved in the results to ensure that our system performed as expected. The post-processing of the YOLOv3 algorithm is realized using the PyTorch library because it is an efficient and lightweight option. Figure 7 illustrates the processing of images by the proposed architecture. After processing using the RRAM PIM, the images are effectively identified. The architecture parameters of the overall simulation are listed in Table 2.

The simulation results are presented in Table 3. In the simulation, the image size is unified to 416 × 416 pixels. The data bit length of the output feature is 16 bit at a working frequency of 50 MHz. A comparison of the operation and memory resource required between this RRAM PIM architecture with those of other architectures is also presented in Table 3.

The image processing speed of the RRAM PIM in this work is approximately 7 times faster than that of the GPU (NVIDIA RTX 3080), i.e., the speeds of the former and the latter are 284 and 39 frames per second (FPS), respectively. This indicates that the RRAM PIM exhibits better performance than the GPU, although its working efficiency is relatively lower than that of the GPU. The RRAM PIM with ADCs proposed by Khwa et al. achieves an image processing speed of 11,606 FPS with ResNet20, which is equivalent to 14.37 FPS with YOLOv3. Therefore, the image processing speed of the RRAM PIM in this work is approximately 20 times faster. The energy consumption and area of ADCs in the RRAM PIM with ADCs proposed by Peng et al. are 12 times and 20 times that of the RRAM arrays, respectively, which can be significantly reduced in the proposed architecture.

Weight replication is used to ensure that each stage of the pipeline requires the same amount of time. Therefore, the RRAM PIM consumes 877.22 Mbit of RRAM resources, which is more than th 497.84 Mbit (quantized to 8-bits) required by the YOLOv3 algorithm. In general, the RRAM PIM outperforms other architectures in terms of the identification rate for heavy-load CNN algorithms, speed, and image size.

The data type of images after normalization is floating point. When the image data are mapped from floating point to fixed point, the data bit width is changed, which may affect the accuracy of the accelerator. To evaluate the overall accuracy of the accelerator, 5000 images with a size of 416 × 416 pixels from the COCO dataset were used for the examination.

**Figure 8:**
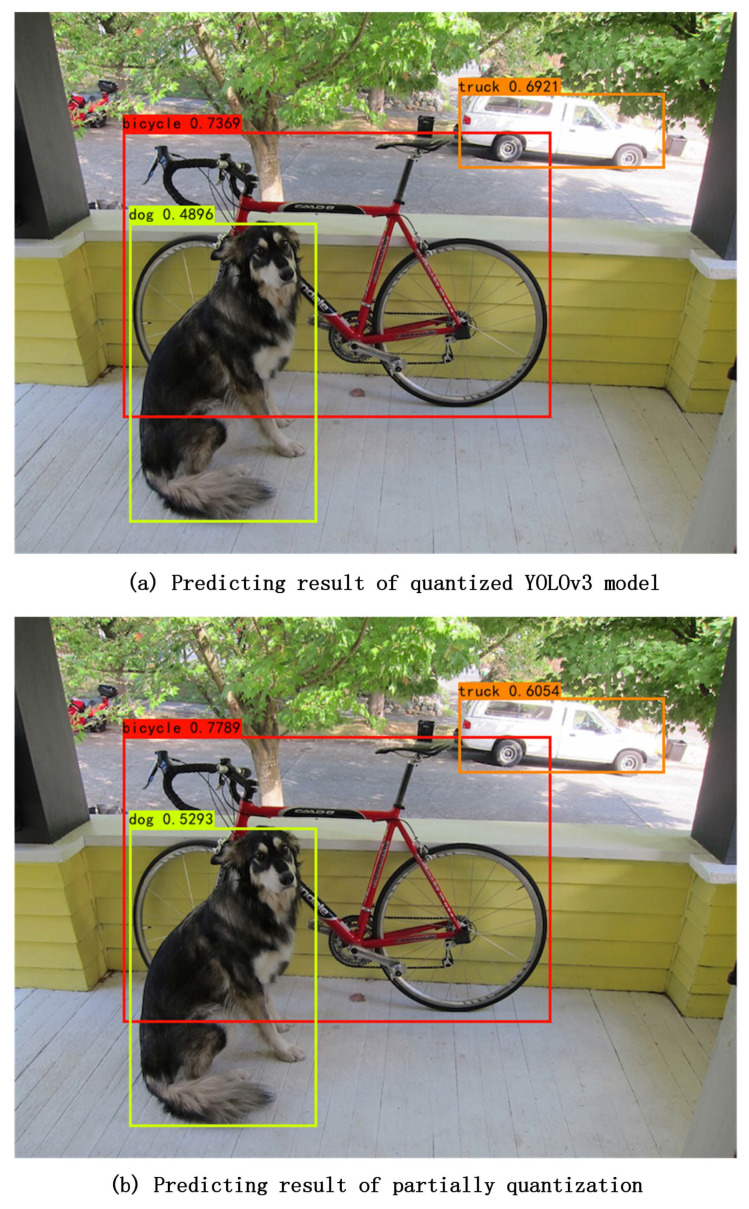
Comparison of predicting results between the fully quantized model and the partially quantized model.

Figure 8 compares the prediction results of the fully quantized model and the partial quantized model proposed in this work, based on a typical prediction in a single picture using the YOLOv3 model. As shown in Figure 8, both models accurately predict the presence of a dog, a bicycle, and a truck. In terms of the predicting precisions, the partially quantized model outperforms the then fully quantized model, with the exception of the truck. This is a situation existing in all of the prediction series in pictures. Although the quantized model may not show better precision in a specific series, the partial quantized model has a better mean Average Precision (mAP).

**Figure 9:**
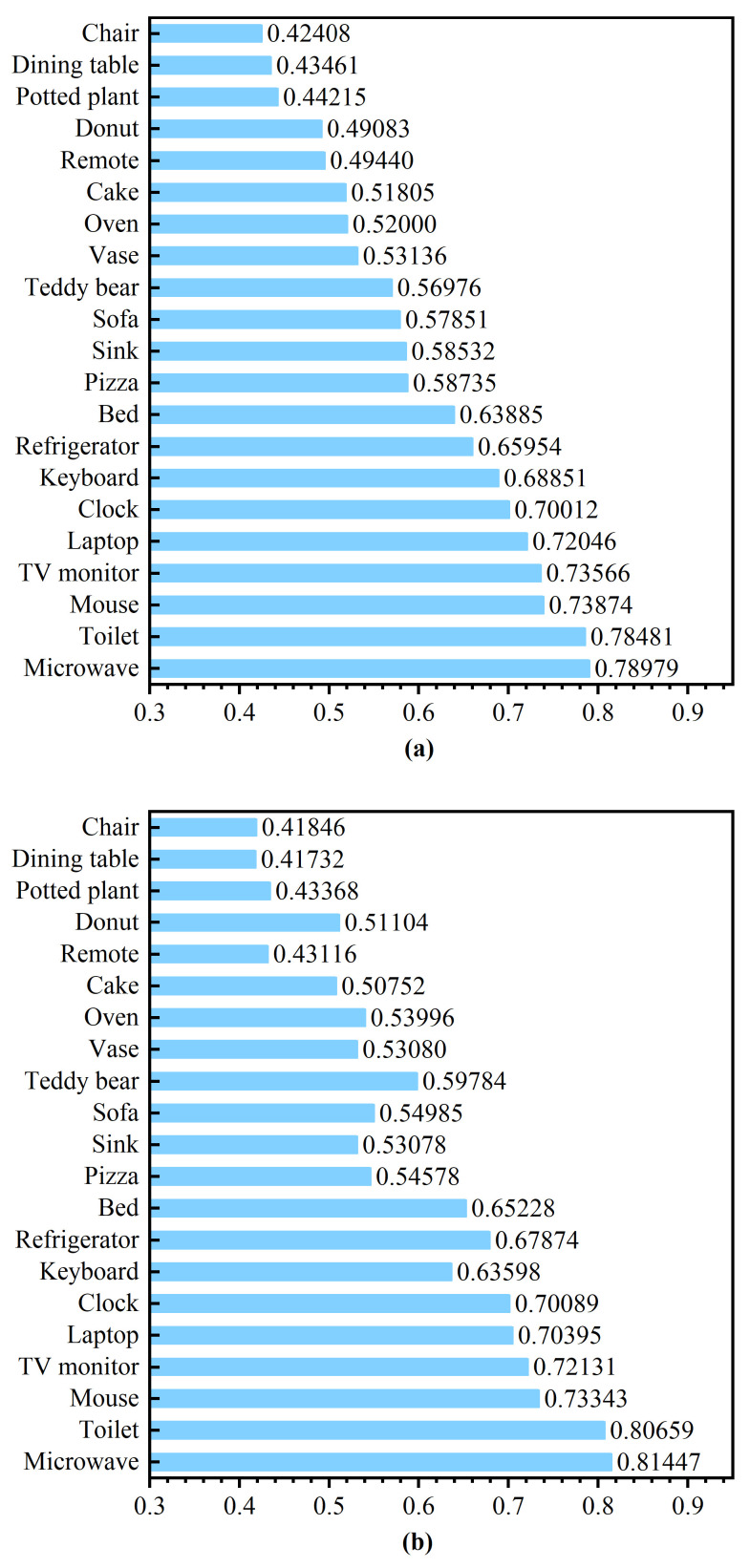
(**a**) Average precision without quantization and (**b**) average precision of partial quantization for different series.

Figure 9 illustrates the average precision of the partially quantized model and that of the model without quantization for different prediction series. The results show that, for most series, the average precision of the partially quantized model only slightly decreases compared to the model without quantization. For some specific series, the partially quantized model performs even better than the model without quantization. That means that the proposed partial quantization approach can effectively reduce the computation resources in the RRAM PIM.

**Figure 10:**
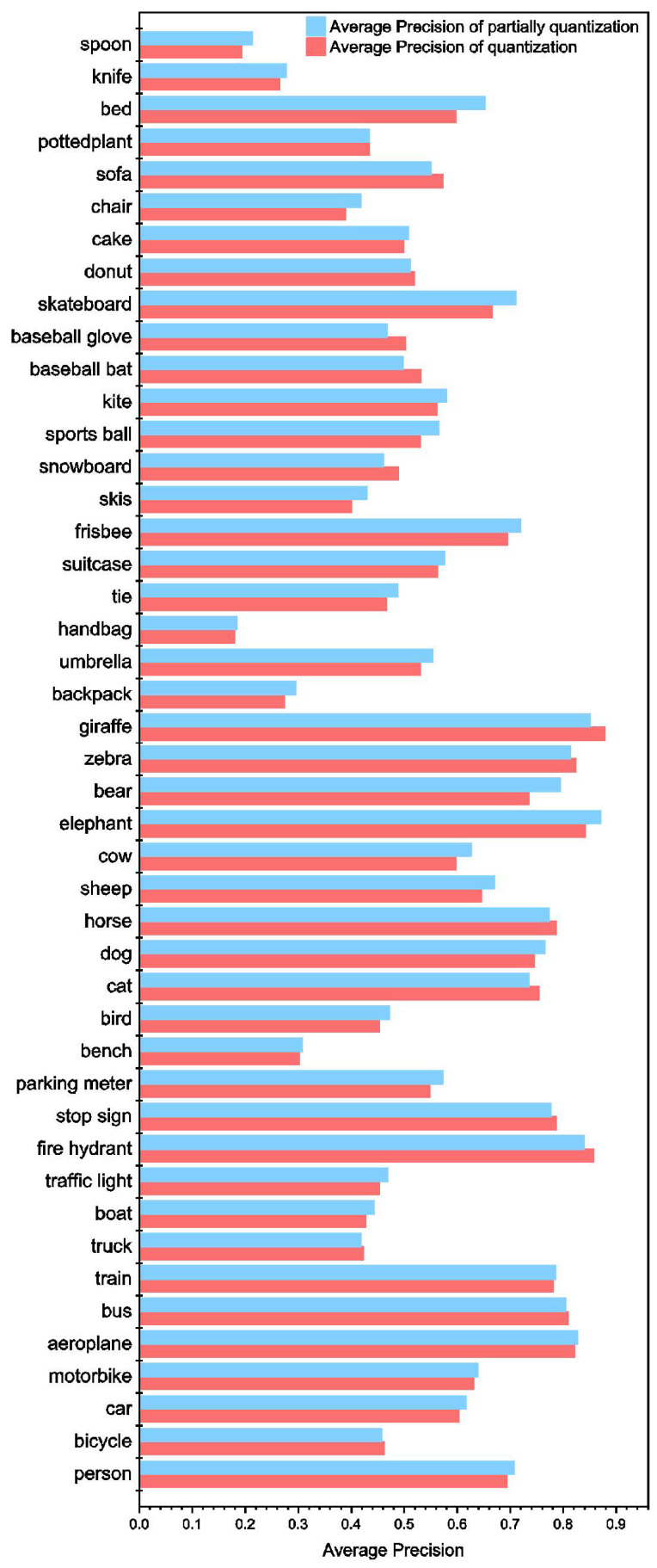
Comparison of average precision between fully quantized model and the partial quantized model for different series in COCO database.

**Figure 11:**
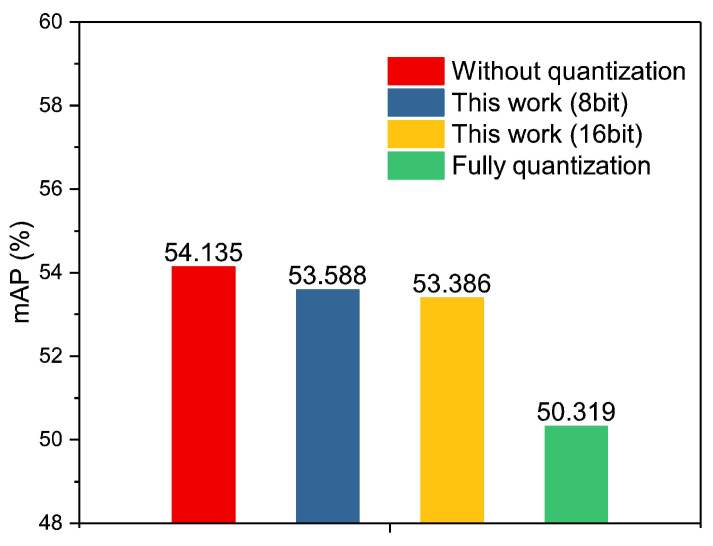
Comparison of the accuracy of the proposed RRAM PIM among different quantization schemes.

Figure 10 illustrates the comparison of average precision between the fully quantized model and the partially quantized model for different series in the COCO database. As depicted in Figure 10, the model with partial quantization exhibits better average precision in most of the series, but not for all. Hence, it achieves a better overall mAP for all series. The mAP for various quantization techniques is presented in Figure 11. As can be observed in the figure, the mAP of the proposed RRAM PIM without quantization is 54.135%, while that of partial quantization with 8-bit and 16-bit are 53.588% and 53.386%, respectively. Compared with the mAP without quantization, the mAP of the partial quantization with 16-bit or 8-bit precision exhibits an insignificant decrease. However, the mAP of full quantization is 50.319%, which causes a 4% decrease in mAP.

## 4. Conclusions

In this work, an RRAM PIM architecture is designed to accelerate CNN networks to achieve an ultra-high frame rate. A partial quantization technique is proposed to reduce the accuracy loss. The YOLOv3 algorithm is used to build a system model for simulation. The resulting image recognition speed reaches 284 FPS, which is approximately 7 times faster than the Nvidia RTX 3080 GPU. The proposed partial quantization techniques, with 8-bit and 16-bit precision, achieved accuracies of 53.588% and 53.386%, respectively. These accuracies of partial quantization remained almost unchanged compared with the algorithm without quantization. 

## Figures and Tables

**Table 1:** The parameters of Au/Ni/${\mathrm{HfO}}_{2}$/Ni RRAM model.

$R_{ON}$	$R_{OFF}$	$V_{ON}$	$V_{OFF}$	τ	*T*	β
245	$2.45\times 10^{7}$	3.2	2.45	$10^{-5}$	298.5	−500

**Table 2:** The architecture parameters of the overall simulation.

Algorithm	Process	Operation Voltage	Working Frequency	RRAM Resource	Feature
YOLOv3	SMIC-55 nm	1.2 V	50 MHz	877.22 MBit	782.52 MBit

**Table 3:** Comparison of operation and memory resource among different architectures.

	FeFET PIM [27]	PIM on FPGA [28]	GPU	RRAM PIM with ADCs by khwa et al. [29]	RRAM PIM with ADCs by Peng et al. [21]	This Work
Algorithm	LeNet-5	YOLOv3-Tiny	YOLOv3	ResNet20	ResNet34	YOLOv3
Accelerator	FeFETs PIM	BRAM PIM	Nvidia RTX 3080	RRAM	RRAM	RRAM
Speed	20 FPS	91.7 FPS	39 FPS	11,606 FPS	132,476 FPS	284 FPS
Resource	\	69.9 KB LUT, 9.3 KB LUTRAM	10 GBit DDR	2 MB RRAM	780 MBit RRAM	877.22 MBit RRAM
Quantization	8 bit	3 bit	No quantization	8-8-19 bit	8 bit	16 bit/8 bit
Image size	32 × 32	256 × 144	416 × 416	32 × 32	224 × 224	416 × 416
Working frequency	100 Hz	1000 MHz	2100 MHz	62.9 MHz	\	50 MHz

## Data Availability

The data presented in this study are available on request from the corresponding author.
